# Supplementary material for: Conditional chemoconnectomics (cCCTomics) as a strategy for efficient and conditional targeting of chemical transmission
Source: eLife. 2024 Apr 30;12:RP91927. doi: 10.7554/eLife.91927 (PMC11060718; doi:10.7554/eLife.91927)
Supplement: Figure 3—source data 3. [file elife-91927-fig3-data3.zip › Figure 3 source data3 part 2.zip!/Figure 3-Source data 3-part2/1052-13-CRISPResso_Report_ejedac2.zip!/Nucleotide_percentage_quilt_around_sgRNA_AGTGGACAAACTGGTACATG.pdf]

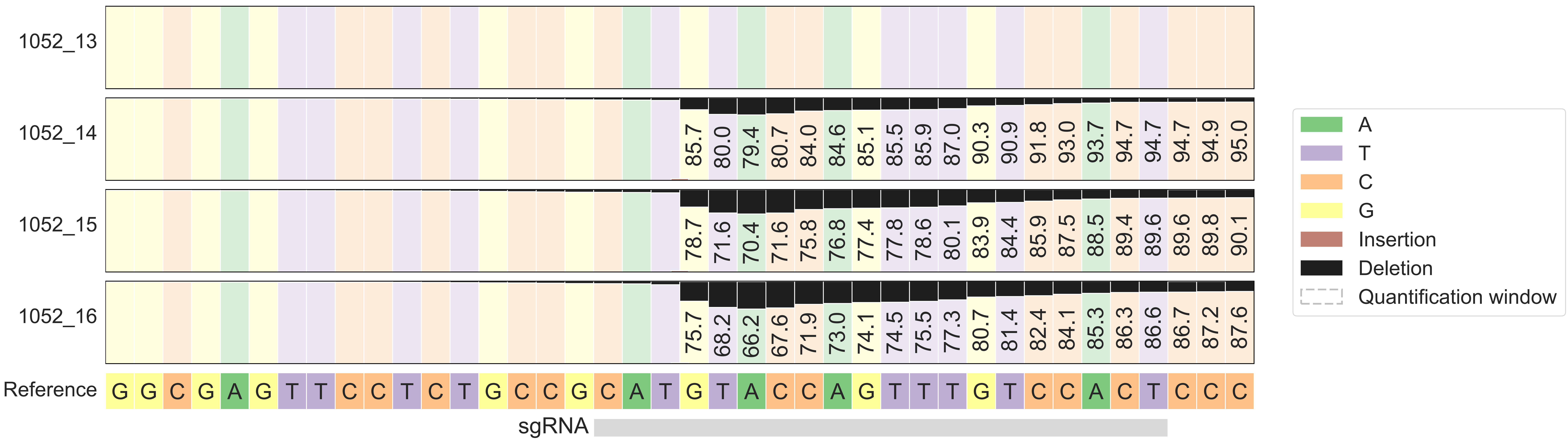

1052_13
1052_14
85.7 80.0 79.4 80.7 84.0 84.6 85.1 85.5 85.9 87.0 90.3 90.9 91.8 93.0 93.7 94.7 94.7 94.7 94.9 95.0
1052_15
78.7 71.6 70.4 71.6 75.8 76.8 77.4 77.8 78.6 80.1 83.9 84.4 85.9 87.5 88.5 89.4 89.6 89.6 89.8 90.1
1052_16
75.7 68.2 66.2 67.6 71.9 73.0 74.1 74.5 75.5 77.3 80.7 81.4 82.4 84.1 85.3 86.3 86.6 86.7 87.2 87.6
Reference
G G C G A G T T C C T C T G C C G C A T G T A C C A G T T T G T C C A C T C C C
sgRNA
A
T
C
G
Insertion
Deletion
Quantification window
